# Supplementary material for: Identification of Immuno-Targeted Combination Therapies Using Explanatory Subgroup Discovery for Cancer Patients with EGFR Wild-Type Gene
Source: Cancers (Basel). 2022 Sep 29;14(19):4759. doi: 10.3390/cancers14194759 (PMC9564073; doi:10.3390/cancers14194759)
Supplement: Supplementary file 1 [file cancers-14-04759-s001.zip › cancers-1892794-supplementary.pdf]

cancers MDPI

Supplementary Materials: Identification of Immuno–Targeted Combination Therapies Using Explanatory Subgroup Discovery for Cancer Patients with EGFR Wild–Type Gene

Olha Kholod, William Basket, Danlu Liu, Jonathan Mitchem, Jussuf Kaifi, Laura Dooley and Chi–Ren Shyu

Table S1. The summary for the proportional odds model.

Predictor	Value	Std. Error	*t* value	*p* value
C1QA	6.89187286	6.7934441	1.0144888	0.3103495776
C1QB	1.52901186	9.4436835	0.1619084	0.8713779735
C1R	−9.00048669	9.1254449	−0.9863066	0.3239826761
C1S	8.71352891	8.3106849	1.0484730	0.2944207248
CDH5	−3.54898803	1.7693896	−2.0057697	0.0448808290
CSF1	−0.62835764	3.0745108	−0.2043765	0.8380593237
CSF1R	2.01424032	1.4266268	1.4118902	0.1579823084
CXCR2	0.42765514	0.2879529	1.4851563	0.1375024129
FCGR1A	−0.29957374	0.3761806	−0.7963561	0.4258250796
FCGR2A	−5.26999148	6.3559740	−0.8291399	0.4070252631
FCGR2B	9.70798109	2.7078036	3.5851866	0.0003368373
FCGR3A	−6.70215788	3.8532025	−1.7393734	0.0819691029
IDO1	2.28734140	1.1896622	1.9226813	0.0545200805
IGF1R	7.71361964	3.8821924	1.9869236	0.0469308638
ITK	−2.06038632	0.7716085	−2.6702485	0.0075795133
JAK2	−11.46642378	5.6822496	−2.0179374	0.0435977859
KIT	1.27297874	0.4280837	2.9736682	0.0029426309
MAP2K2	−10.86452910	8.0887820	−1.3431601	0.1792201462
PBK	−0.08865425	0.3955506	−0.2241287	0.8226571363
PDGFRB	6.87766311	3.8400577	1.7910312	0.0732882892
PSMB10	2.18473680	6.3441690	0.3443693	0.7305686009
PSMB9	−0.89717334	4.1000446	−0.2188204	0.8267899664
PTGS2	−0.75415429	0.4883655	−1.5442415	0.1225298273
PR∣CR	−12.53076667	32.4375601	−0.3863042	0.6992713682
CR∣PD	−12.15702111	32.4333455	−0.3748309	0.7077862086
PD∣SD	−9.06253253	32.4213475	−0.2795236	0.7798430112

Table S2. Proportional odds model odds ratios and confidence intervals for the proportional odds model.

Predictor	OR	2.5 %	97.5 %
C1QA	9.842430e+02	1.623724e−03	5.966127e+08
C1QB	4.613616e+00	4.222440e−08	5.041031e+08
C1R	1.233498e−04	2.106422e−12	7.223225e+03
C1S	6.084677e+03	5.130619e−04	7.216146e+10
CDH5	2.875372e−02	8.965756e−04	9.221493e−01
CSF1	5.334672e−01	1.288485e−03	2.208697e+02
CSF1R	7.495031e+00	4.575373e−01	1.227780e+02
CXCR2	1.533657e+00	8.722124e−01	2.696710e+00
FCGR1A	7.411341e−01	3.545612e−01	1.549182e+00
FCGR2A	5.143654e−03	2.000124e−08	1.322777e+03
FCGR2B	1.644836e+04	8.151472e+01	3.319015e+06
FCGR3A	1.228259e−03	6.448192e−07	2.339600e+00
IDO1	9.848719e+00	9.566155e−01	1.013963e+02
IGF1R	2.238631e+03	1.110336e+00	4.513470e+06
ITK	1.274047e−01	2.807962e−02	5.780693e−01
JAK2	1.047600e−05	1.525653e−10	7.193414e−01
KIT	3.571475e+00	1.543342e+00	8.264815e+00
MAP2K2	1.912471e−05	2.491215e−12	1.468178e+02
PBK	9.151619e−01	4.215069e−01	1.986969e+00
PDGFRB	9.703561e+02	5.227188e−01	1.801334e+06
PSMB10	8.888309e+00	3.537144e−05	2.233498e+06
PSMB9	4.077205e−01	1.319466e−04	1.259873e+03
PTGS2	4.704083e−01	1.806248e−01	1.225102e+00
